# A study on the incremental value of soothing moisturizing repairing cream on dupilumab in children with severe atopic dermatitis

**DOI:** 10.1097/MD.0000000000045078

**Published:** 2026-01-30

**Authors:** Yangyang Lin, Zhiwei Guan, Xinxin Liu, Yuantao Song, Huangman Xiao, Liang Yin, Qinfeng Li

**Affiliations:** a The Department of Dermatology, Tianjin Children's Hospital (Children's Hospital, Tianjin University), Tianjin Key Laboratory of Brith Defects for Prevention and Treatment, Tianjin, P.R. China; b Department of Nuclear Medicine, Pingjin Hospital, Characteristic Medical Center of Chinese People’s Armed Police Forces, Tianjin, P.R. China.

**Keywords:** atopic dermatitis, children, dupilumab, severe, soothing moisturizing repair cream

## Abstract

Soothing Moisturizing Repair Cream is a newly launched emollient with anti-inflammatory, itch-relieving, and moisturizing properties. The purpose of this study is to evaluate the incremental value of Soothing Moisturizing Repair Cream in the efficacy of dupilumab monotherapy for children with severe atopic dermatitis (AD). This study included 36 children with severe AD who completed a 12-week treatment regimen from November 2022 to April 2023. There were 17 children in the experimental group and 19 in the control group. The control group received only dupilumab monotherapy, while the experimental group used Soothing Moisturizing Repair Cream plus dupilumab. Changes in Scoring of AD (SCORAD), AD Control Tool (ADCT), and Numerical Rating Scale (NRS) scores before and after treatment were compared, along with any adverse reactions to soothing moisturizing repairing cream. There were no significant differences in gender, age, disease duration, cumulative dose of dupilumab, SCORAD, ADCT, and NRS scores between the 2 groups before treatment. Both groups showed a gradual decline in SCORAD, ADCT, and NRS scores at 2, 4, 8, and 12 weeks after starting dupilumab treatment, with significant differences between each time point (*P* < .05). The experimental group showed further significant reductions in SCORAD, ADCT, and NRS scores compared to the control group at the same time points (*P* < .05). Adverse reactions to were generally mild. Our real-life data seems to confirm the Moisturizing Repair Cream significantly enhances the effectiveness of dupilumab monotherapy in treating children with severe AD.

## 1. Introduction

Atopic dermatitis (AD) is a chronic, recurrent inflammatory skin disease that predominantly affects children, with most cases arising in infancy. The prevalence in adults is approximately 2% to 8%, whereas in children it is as high as 10% to 20%.^[1,2]^ The disease is characterized by dry skin, persistent itching, and polymorphic skin lesions, often showing a familial genetic predisposition. The condition tends to relapse frequently and can be prolonged and unresolved, significantly impacting patients’ quality of life.^[3,4]^ Poorly controlled severe AD in children can lead significantly reduced quality of life for both patients and their families, impair growth and development, and add to increased health care costs.^[5–7]^

Traditional treatments have been less than satisfactory for severe AD. Dupilumab injection, a novel biologic, was first approved monoclonal antibody targeting type 2 inflammation, for adolescent and adult patients with moderate-to-severe AD,^[8]^ it had also been approved in 2020 by China’s National Medical Products Administration for the treatment of moderate-to-severe AD, dupilumab demonstrated satisfactory efficacy in patients aged ≥ 6 years with moderate-to-severe AD, as well as in other type 2 inflammatory diseases.^[9–15]^ Considering the unique value of emollients in AD treatment, Soothing Moisturizing Repair Cream, a newly launched emollient with anti-inflammatory, itch-relieving, and moisturizing properties. In this study, we enrolled patients aged 0 to 6 and 6 to 12 years old to explore the dupilumab efficacy and the incremental value of Soothing Moisturizing Repair Cream combined with dupilumab monotherapy.

## 2. Materials and methods

### 2.1. Study design

From November 2022 to April 2023, children with severe AD were recruited from the department of dermatology of our hospital. Based on the primary endpoint (SCORAD score change), a 2-sample t-test was performed with target effect size: Δ ≥ 10 points (MCID threshold per EADV consensus*), and standard deviation: σ=12.0 points (derived from Schmitt et al, JACI 2007**), 2-sided α = 0.05, power = 0.80. This yielded 17 subjects per group, accounting for 20% anticipated dropout rate, 20 participants were allocated to each intervention and control group. So, a total of 40 children with severe AD were enrolled in our study, Ethical Statement and research protocol of this study were reviewed and approved by the Ethics Committee of the Hospital (NO. KY2022-31). This study performed in accordance with the Declaration of Helsinki of 2008 and Good Clinical Practice guidelines. Written informed consent was obtained from their legally guardians/parents.

**Figure 1.**
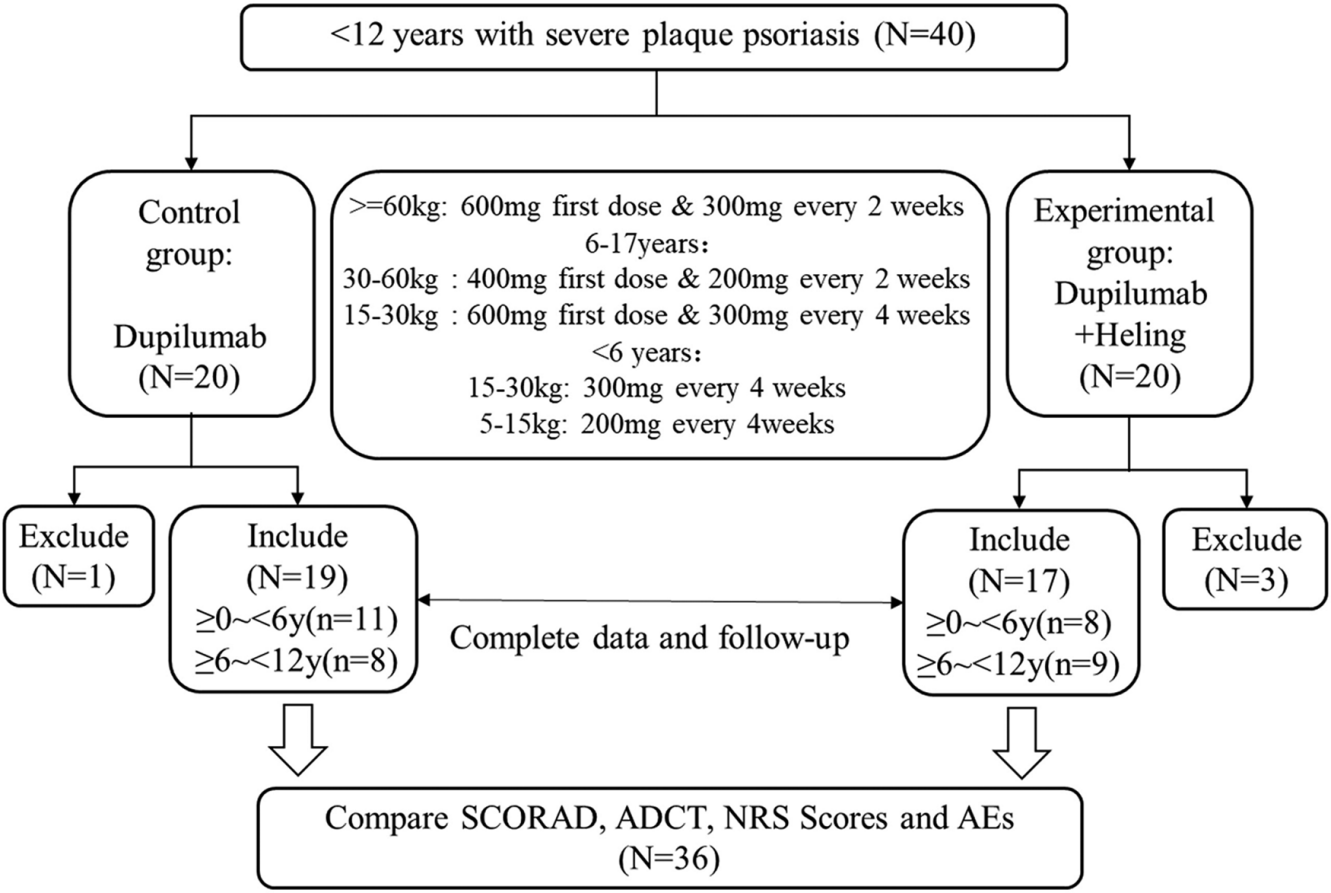
Study workflow and patient disposition.

Patients were simply randomly divided into 2 groups: a control group and an experimental group, with 20 children in each group. The control group treated with involved exclusive administration of dupilumab monotherapy [Brand name: Dupixent, 300 mg (2.0 ml) per prefilled syringe, manufactured by Sanofi (China) Investment Co., Ltd., National Medical Products Administration Approval Number S20200017]. No concurrent oral or injectable medications were used during the treatment period. The dosage of dupilumab injection^[16]^ was listed in Figure 1. In addition to the treatment of dupilumab injection, participants in the experimental group also applied Soothing Moisturizing Repair Cream [150g/tube, manufactured by Pharmaceuticals (Guangzhou) Co., Ltd.] over their entire body after cleaning and drying the affected area by gentle rubbing to facilitate absorption twice daily. Skin hygiene maintenance and avoiding irritants were also advised to patients or their guardians in both control and experimental groups. This study was an open-label trial, no blinding for participants, caregivers, and outcome assessors, employed a modified intention-to-treat (mITT) population as the primary analysis set (intervention n = 17/control n = 19). Missing data from dropouts (15% intervention/5% control) were handled using multiple imputations supplemented by worst-case sensitivity analysis.

### 2.2. Inclusion, exclusion and termination criteria

Inclusion criteria: conforming to the diagnostic criteria for AD as outlined in the “Guidelines for the Diagnosis and Treatment of AD in China (2020 Edition)”^[17]^ or meeting Williams’ diagnostic criteria; aged ≥ 3 years, capable of clearly expressing symptoms of itching and pain, with no gender restriction; SCORAD score > 50; consent of the child’s guardian to participate in this clinical observation, to follow treatment as directed by the physician throughout the study period, and to sign an informed consent form. Exclusion criteria: skin lesions with trauma, infection, or significant exudative tendency; systemic use of corticosteroids or immunosuppressants in the past 2 weeks; allergy to any of the medications or skincare products used in the clinical observation; noncompliance with medical advice, violation of the treatment plan, loss to follow-up, or incomplete data; history of liver or kidney dysfunction, hematological disorders, or cancer; recent history of helminth infection or vaccination with a live attenuated vaccine within the past 4 weeks or anticipated during the trial period.

### 2.3. Efficacy indicators

Follow-up observations were conducted at the end of 2, 4, 8, and 12 weeks of treatment. Photographs were taken to record and evaluate the improvement in the children’s rash, itching, and subjective symptoms. Primary evaluation metrics included: the SCORAD to assess changes in the severity of skin lesions before and after treatment; and the atopic dermatitis control tool (ADCT) to evaluate the degree of disease remission before and after treatment; and the numerical rating scale (NRS) to assess changes in the severity of itching before and after treatment.

### 2.4. Safety assessment

Adverse events (AEs) caused by medication use in both groups were also observed.

## 3. Statistical analysis

Data was analyzed using the SPSS 26.0 statistical software package. Categorical data were presented as percentages (%) and analyzed using the *χ*^*2*^ test in R × C contingency tables. Quantitative data, conforming to a normal distribution, was expressed as mean ± standard deviation (±SD). Comparisons of scores before and after treatment were conducted using paired-sample t-tests or analysis of variance for repeated measures data. A *P*-value of < 0.05 was considered statistically significant.

## 4. Results

### 4.1. Study population and patient disposition

Overall, 36 children had completed the 12-week treatment and follow-up, including 17 in the experimental group and 19 in the control group. The remaining 4 children were excluded from the study due to noncompliance with the treatment and follow-up protocol. Among the 36 children, there were 20 males and 16 females, with an average age of 7.0 ± 3.0 years (range 3–15 years). The duration of the disease was 42.7 ± 21.4 months (range 16–120 months), and the cumulative dosage of dupilumab was 1494.4 ± 352.1 mg (range 800–2400 mg). Normality was confirmed for all continuous variables using the Shapiro–Wilk test (all *p* > 0.05), justifying the application of parametric methods. The mean SCOARD score was 77.0 ± 12.0 and 76.9 ± 13.2 in experimental and control group, for the ADCT score before treatment was 18.6 ± 2.9 in experimental group and 17.9 ± 2.6 in control group, and the NRS was 8.2 ± 1.4 and 8.3 ± 1.4 in experimental and control group. There were no statistically significant differences between the 2 groups in terms of gender distribution, age, duration of the disease, and cumulative dosage of dupilumab in Table 1.

**Table 1:** Demographic and clinical characteristics of AD patients.

Clinical characteristics	Control (n = 19)	Experimental (n = 17)	*P*
Age (year), mean ± SD			
≥0–<6 yr	5.10 ± 1.04	4.63 ± 1.06	.435
≥6–<12 yr	9.13 ± 1.96	8.44 ± 1.42	.442
Weight(kg), mean ± SD			
≥0–<6 yr	24.36 ± 4.72	21.88 ± 4.09	.247
≥6–<12 yr	42 ± 13.66	38.33 ± 9.58	.527
AD onset (m), mean ± SD			
≥0–<6 yr	31.46 ± 9.05	36.5 ± 12.40	.318
≥6–<12 yr	57.25 ± 22.70	48.89 ± 29.06	.523
Dupilumab dosage (mg), mean ± SD			
≥0–<6 yr	1327.27 ± 228.43	1237.5 ± 238.67	0.418
≥6–<12 yr	1775 ± 388.22	1677.78 ± 272.85	0.555
SCORAD score, mean ± SD			
≥0–<6 yr	82 ± 14.30	78.60 ± 12.7	0.602
≥6–<12 yr	70 ± 7.65	75.60 ± 12.0	0.280
ADCT score, mean ± SD			
≥0–<6 yr	19.50 ± 2.93	18.82 ± 2.18	0.567
≥6–<12 yr	17.89 ± 2.80	16.75 ± 2.77	0.413
NRS score, mean ± SD			
≥0–<6 yr	8.82 ± 0.98	8.38 ± 1.30	0.408
≥6–<12 yr	7.63 ± 1.69	8.11 ± 1.54	0.543

AD = atopic dermatitis, ADCT = atopic dermatitis control tool, NRS = numerical rating scale, SCORAD = scoring of atopic dermatitis.

### 4.2. Clinical efficiency

**Figure 2.**
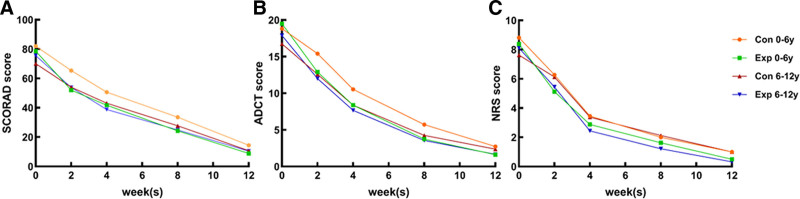
Mean values of SCORAD (A), ADCT (b) and NRS (c) pre- and post-dupilumab treatment in children with AD. Statistical significance was assessed by The Mann–Whitney test and Fisher test: *P < .005, **P < .001, ***P < .0001. AD = atopic dermatitis, ADCT = atopic dermatitis control tool, NRS = numerical rating scale, ns = not significant, SCORAD = scoring of atopic dermatitis.

After 2, 4, 8, and 12 weeks of treatment, the SCORAD, ADCT, and NRS scores of children in both the experimental and control groups (Fig. 2) showed a gradual downward trend over time, with significant differences between the 2 groups at each adjacent time point (*P* < .05). At any given point after treatment, the scores for SCORAD, ADCT, and NRS in the experimental group were consistently lower than those in the control group during the same period, with statistically significant differences (*P* < .05). See Table 2 for details.

**Table 2:** Overall and age subgroup’s severity and pruritus scores of AD patients.

Efficiency	Group	Baseline	2 wk	4 wk	8 wk	12 wk
SCORAD	Control	76.9 ± 13.2	60.8 ± 11.6	47.5 ± 11.5	31.1 ± 8.5	12.9 ± 4.9
	≥0–<6 yr	82.0 ± 14.3	65.8 ± 12.4	50.6 ± 13.4	33.5 ± 8.8	14.5 ± 5.2
	≥6–<12 yr	70.0 ± 7.7	54.0 ± 5.9	43.1 ± 6.5	27.8 ± 7.3	10.6 ± 3.7
	Experimental	77.0 ± 12.0	52.9 ± 9.8	40.2 ± 8.2	24.6 ± 8.1	9.7 ± 4.2
	≥0–<6 yr	78.6 ± 12.7	52.0 ± 10.5	41.8 ± 10.7	24.3 ± 6.5	8.9 ± 3.4
	≥6–<12 yr	75.6 ± 12.0	53.8 ± 9.8	38.8 ± 5.7	24.9 ± 9.7	10.4 ± 4.9
ADCT	Control	17.9 ± 2.6	14.2 ± 2.6	9.6 ± 2.3	5.1 ± 2.0	2.6 ± 1.2
	≥0–<6 yr	18.8 ± 2.2	15.3 ± 2.4	10.6 ± 2.2	5.7 ± 2.3	2.7 ± 1.5
	≥6–<12 yr	16.8 ± 2.8	12.6 ± 2.1	8.4 ± 2.0	4.3 ± 1.0	2.4 ± 0.7
	Experimental	18.6 ± 2.9	12.4 ± 2.1	8.0 ± 2.1	3.6 ± 1.7	1.6 ± 0.9
	≥0–<6 yr	19.0 ± 2.9	12.9 ± 2.1	8.4 ± 2.5	3.8 ± 2.3	1.6 ± 0.9
	≥6–<12 yr	17.9 ± 2.8	12.0 ± 2.2	7.7 ± 1.7	3.6 ± 1.2	1.7 ± 0.8
NRS	Control	8.3 ± 1.4	6.2 ± 1.2	3.4 ± 1.2	2.1 ± 0.9	1.0 ± 0.7
	≥0–<6 yr	8.8 ± 1.0	6.3 ± 1.3	3.5 ± 1.0	2.0 ± 1.1	1.0 ± 0.8
	≥6–<12 yr	7.6 ± 1.7	6.1 ± 1.1	3.4 ± 1.4	2.1 ± 0.6	1.0 ± 0.5
	Experimental	8.2 ± 1.4	5.3 ± 1.1	2.6 ± 1.0	1.4 ± 0.9	0.4 ± 0.5
	≥0–<6 yr	8.4 ± 1.3	5.1 ± 1.0	2.9 ± 1.1	1.6 ± 1.1	0.5 ± 0.5
	≥6–<12 yr	8.1 ± 1.5	5.4 ± 1.3	2.4 ± 0.9	1.2 ± 0.7	0.3 ± 0.5

AD = atopic dermatitis, ADCT = atopic dermatitis control tool, NRS = numerical rating scale, SCORAD = scoring of atopic dermatitis.

### 4.3. AEs assessment

Participants did not receive any test dose of dupilumab before the formal trial. No moderate or severe drug-related adverse reactions occurred in either group. In the experimental group, 1 child developed a localized allergic reaction at the site of Soothing Moisturizing Repair Cream application, with an incidence rate of 5.9%. Additionally, 2 children in each group experienced adverse reactions related to dupilumab, in the experimental group, these included 1 case of injection site reaction and 1 case of conjunctivitis, while in the control group, there was 1 case of headache and 1 case of injection site reaction.

## 5. Discussion

The pathogenesis of AD is complex and not yet fully understood, potentially resulting from a combination of factors such as genetic predisposition, skin barrier defects, immune dysregulation, microbial infection, environmental factors, diet, and negative emotional states.^[17,18]^ Persistent itching is the most prominent subjective symptom in children with AD. Traditional treatment methods for AD include the use of emollients, corticosteroids, calcineurin inhibitors, oral antihistamines, and phototherapy. Sole reliance on traditional moisturizers has proven ineffective. Long-term corticosteroid use can lead to side effects such as skin atrophy and telangiectasia. Additionally, the potential for adverse reactions like burning or stinging with calcineurin inhibitors limits their use.

The highly pruritic and often stigmatizing skin lesions may lead to sleep deprivation, poor school performance, low self-esteem, and familial stress. The risk of developing psychiatric co-morbidities, such as depression and anxiety disorders, is significantly increased in patients being severely affected by AD.^[19]^

In recent years, biologics have begun to be used in the clinical treatment of AD. Dupilumab is a fully human monoclonal antibody that inhibits the signaling of IL-4 and IL-13, thereby blocking the inflammation they mediate.^[20]^ Clinical trial data from abroad indicates that the level of evidence for dupilumab in treating moderate-to-severe AD is 1a, with a recommendation strength of A^4^. In a Phase 2 clinical trial involving children aged 6 months to 6 years with severe AD, a single dose of dupilumab significantly improved clinical symptoms and signs of AD, with the itch index decreasing by 22.9% to 44.7% after 3 weeks of treatment.^[21,22]^

The most common side effects were injection site reactions and conjunctivitis, which are both known from the adult studies,^[23]^ and the risk of ocular complications did not correlate with dupilumab in a dose-dependent manner.^[15]^

Previous publications of dupilumab clinical trials also reported that dupilumab treatment was associated with increased incidence of conjunctivitis.^[23,24]^ A long-term observational study from the BioDay Registry in the Netherlands (210 patients) indicated that patients receiving dupilumab also reported unspecific pain symptoms: 1) headache in 9.4% of cases; 2) muscle or joint pain in 7.6% of the cases, and in 1 case dupilumab had to be even discontinued due to muscle pain.^[22]^ Research in our study shows that dupilumab is highly effective in treating AD and does not have significant serious adverse reactions.

Dupilumab rapidly improves Peak Pruritus, anxiety, depression and quality of life by week 2. At week 16, more dupilumab-treated than placebo-treated patients reported improvement in SCORAD itch and sleep.^[25]^

Our study demonstrates that treatment with dupilumab alone in the control group also can achieve noticeable effects within 2 weeks, reducing SCORAD, ADCT, and NRS scores. Subsequently, these scores showed a gradual decreasing trend over time, consistent with the results reported in the literature.

Emollients are a fundamental part of the stepwise treatment for AD, with their crucial role in AD management widely recognized in relevant guidelines and consensuses. Use of skincare products with skin barrier repair and moisturizing functions can alleviate symptoms such as skin dryness and itching in AD patients.

Soothing Moisturizing Repair Cream we use in this study is an emollient containing a blend of active ingredients including 4-tert-butylcyclohexanol, avocado fruit extract, licorice root extract, and saccharide isomers. According to prior research, these ingredients have strong antihistamine and anti-inflammatory effects, which can soothe sensitive skin and relieve stinging and itching, improving skin barrier function.^[26,27]^

In this study, Soothing Moisturizing Repair Cream was introduced as an intervention factor. The results indicate that for children with severe AD, the combined use of Cream with standard dupilumab therapy is more effective than dupilumab monotherapy. This combination further reduces the SCORAD, ADCT, and NRS scores of the children, with the effectiveness increasing over time, demonstrating a significant therapeutic additive value.

Additionally, in the experimental group, only 1 child experienced a localized allergic reaction at the site of cream application, indicating a low and mild incidence rate of adverse reactions to Cream. Both groups experienced 2 minor adverse reactions caused by dupilumab, which resolved without special treatment and disappeared upon observation, consistent with findings reported in literature.^[23]^

Subgroup analyses in this study showed that the overall efficacy of dupilumab may be better in the toddler group of 0 to 6 years old, and in the child group of 6 to 12 years old, Overseas studies have also reported that the improvement in the efficacy indexes of dupilumab in children with AD is more pronounced than that in adults, with the younger age group having a better response than the older age group. This may be related to the more pronounced Th2-type inflammation and more allergic co-morbidities in pediatric AD.^[28]^

## 6. Study limitations

However, our study has several limitations, Firstly, the patients included were limited to Chinese children, future research involving diverse ethnicities were needed. Secondly, the duration of treatment in our study was relatively short. Thirdly, our study was conducted with a limited number of children at a single center, subsequent studies will aim to include comprehensive analyses of data across various age groups and multicenter collaborations.

## 7. Conclusion

In summary, dupilumab demonstrated significant efficacy and a favorable safety profile in clinical trials, and Soothing Moisturizing Repair Cream significantly enhances the efficacy of dupilumab in treating children with severe AD, and its use is straightforward and safe.

## Author contributions

**Conceptualization:** Yangyang Lin, Zhiwei Guan, Xinxin Liu, Liang Yin, Qinfeng Li.

**Data curation:** Yangyang Lin, Zhiwei Guan.

**Formal analysis:** Yangyang Lin, Huangman Xiao.

**Funding acquisition:** Qinfeng Li.

**Investigation:** Yangyang Lin, Yuantao Song, Liang Yin.

**Methodology:** Xinxin Liu, Yuantao Song, Huangman Xiao.

**Project administration:** Huangman Xiao.

**Resources:** Xinxin Liu.

**Software:** Xinxin Liu, Yuantao Song.

**Supervision:** Xinxin Liu, Yuantao Song.

**Validation:** Yuantao Song.

**Visualization:** Xinxin Liu, Liang Yin.

**Writing – original draft:** Yangyang Lin, Zhiwei Guan, Yuantao Song, Liang Yin.

**Writing – review & editing:** Yangyang Lin, Liang Yin, Qinfeng Li.
